# MicroRNAs in Breast Cancer: Diagnostic and Prognostic Potential, Challenges, and Clinical Reliability

**DOI:** 10.3390/biomedicines14030502

**Published:** 2026-02-25

**Authors:** Cara-Xenia-Rafaela Neagoe, Maximilian Gundershausen, Mihaela Ionică, Octavian Constantin Neagoe

**Affiliations:** 1 Doctoral School, “Victor Babes” University of Medicine and Pharmacy, Eftimie Murgu Square No. 2, 300041 Timisoara, Romania; 2 Breast Surgery Research Center, “Victor Babeș” University of Medicine and Pharmacy, 300079 Timișoara, Romania; maximilian.gundershausen@student.umft.ro (M.G.);; 3 Second Clinic of General Surgery and Surgical Oncology, Emergency Clinical Municipal Hospital, 300079 Timișoara, Romania; 4 Second Discipline of Surgical Semiology, First Department of Surgery, “Victor Babeș” University of Medicine and Pharmacy, 300041 Timișoara, Romania

**Keywords:** miRNA, breast cancer, liquid biopsy, cancer proliferation, cancer metastasis, diagnostic biomarkers, prognostic biomarkers, drug resistance, therapeutic targets

## Abstract

Despite the rise in precision medicine, breast cancer management still lacks the non-invasive tools necessary to track tumor dynamics in real time. MicroRNAs (miRNAs) have emerged as strong candidates to fill this gap, particularly within the liquid biopsy framework. Their inherent stability in circulation and their ability to reflect specific molecular changes make them compelling biomarkers for clinical use. This review outlines the current state of miRNA research in breast cancer, specifically assessing their utility in early diagnosis and the prediction of patient outcomes. The focus is on a range of high-priority targets, such as miR-21, miR-155, and the miR-200 family, which have demonstrated consistent dysregulation across different molecular subtypes of breast cancer. These molecules offer a distinct advantage over traditional protein markers by providing a more precise look at tumor progression and therapeutic resistance. However, the transition from discovery to clinical practice remains blocked by technical inconsistencies. The lack of standardized protocols for RNA isolation and the difficulty in identifying reliable reference genes for normalization continue to affect reproducibility. While the potential for these biomarkers is well-documented, the field must now shift its focus toward establishing clinical reliability. Large-scale prospective validation studies are on the horizon to facilitate this implementation. All in all, international consortia and multi-center trials are required to test circulating miRNA biomarkers in real-world settings, ensuring they are feasible enough to guide routine oncological decision-making.

## 1. Introduction

Breast cancer represents a significant focus within oncology, surgery, and pathology due to its status as the most prevalent site-specific malignancy among the female population. Global epidemiological data from 2022 indicates that the incidence of breast malignancy has surpassed that of lung cancer, establishing it as the most frequently diagnosed primary malignancy worldwide [1].

The vast majority of breast malignancies are classified as adenocarcinomas, predominantly originating within the terminal duct lobular unit (TDLU). These neoplasms are further categorized as ductal or lobular carcinomas based on the specific anatomical and histological structures from which the malignancy arises [2,3,4,5].

In clinical practice, the adoption of molecular classification—specifically surrogate molecular subtyping via immunohistochemistry (IHC)—is essential for therapeutic stratification. These molecular profiles serve critical prognostic and predictive functions, particularly in determining the likelihood of tumoral response to endocrine and targeted therapies [6]. The identification of these subtypes relies on the expression of estrogen receptor (ER), progesterone receptor (PR), human epidermal growth factor receptor 2 (HER2), and the proliferation marker Ki-67 [5,7,8].

The primary determinant of breast cancer survival is the clinical stage at the time of identification. A critical distinction exists between screen-detected and symptom-detected malignancies; research indicates that the odds of being diagnosed with advanced-stage disease (Stage IIA or greater) are 6.60 times higher for symptom-detected cases compared to those identified through systematic screening [9].

In the context of oncogenesis and tumor progression, microRNAs (miRNAs) exert an essential regulatory role. Breast cancer is characterized by profound genetic and clinical heterogeneity. The unique miRNA expression profiles observed in these malignancies are disease-specific and vary significantly across molecular subtypes, suggesting that these signatures are critical determinants of clinical behavior and potential therapeutic vulnerability [10,11].

While traditional tissue biopsy remains the gold standard for initial diagnosis, its limitations regarding tumor heterogeneity are increasingly evident. Breast cancer exhibits significant intra-tumor heterogeneity, thus discrete regions of the same primary tumor manifest distinct genetic and metabolic profiles [10].

The pursuit of minimally invasive diagnostic strategies has brought the concept of liquid biopsy to the forefront of clinical research. This technique utilizes circulating miRNAs (c-miRNAs) found in biofluids, such as serum or plasma, as non-invasive biomarkers. MicroRNAs offer several compelling advantages in this context. Primarily, they facilitate non-invasive testing; unlike tissue biopsies that necessitate surgical intervention or needle extraction, c-miRNAs can be quantified from peripheral blood draws, thereby minimizing patient discomfort and procedural risk. This non-invasiveness facilitates serial sampling, allowing for longitudinal monitoring and the early detection of molecular shifts. In clinical management, blood-based miRNA assays could be integrated into regular follow-up protocols to complement diagnostic imaging, potentially identifying molecular evidence of relapse prior to symptomatic or radiological manifestation. To date, more than 3000 microRNAs have been identified across nearly all human biofluids, serving as ubiquitous regulators of physiological and pathological states. Due to their extensive capacity to modulate multi-gene expression patterns, miRNAs play a major role in the progression or suppression of various diseases. Their influence spans critical biological processes, including tumorigenesis, apoptosis, and embryogenesis, as well as cell proliferation and differentiation, marking them as central pillars of cellular homeostasis and disease pathology. In various malignancies, elevated microRNA levels—most notably miR-21, miR-155, and miR-210—serve as potent “oncomiRs” that drive tumor progression by silencing key tumor-suppressor genes. While specific markers like miR-1290 in pancreatic cancer and miR-92a in colorectal cancer offer high-sensitivity diagnostic signatures in biofluids, their overexpression is frequently linked to advanced staging, chemoresistance, and poor survival outcomes across lung and gastrointestinal cancers. These upregulated profiles are increasingly utilized as non-invasive tools for early screening and real-time monitoring of metastatic shifts. Consequently, miRNA biomarkers fulfill the clinical requirement for minimally invasive yet highly informative diagnostics [12,13].

The aim of this scoping review is to emphasize the roles of miRNAs in every aspect of breast cancer management. Extensive research has already documented the dysregulation of microRNAs (miRNAs) in breast cancer, yet existing reviews frequently prioritize molecular biogenesis or therapeutic applications over clinical implementation. While these works offer valuable catalogs of miRNA signatures, they often overlook the pronounced variability found across diverse clinical cohorts. This review diverges from traditional descriptive summaries by prioritizing clinical reliability. We move beyond simple identification to critically analyze the methodological barriers—including pre-analytical liquid biopsy inconsistencies and the absence of standardized normalization—that hinder the transition from discovery to the clinic. By integrating diagnostic precision with long-term prognostic stability, this work establishes a rigorous framework for moving miRNA biomarkers into standardized oncological care.

## 2. Materials and Methods

**Figure 1:**
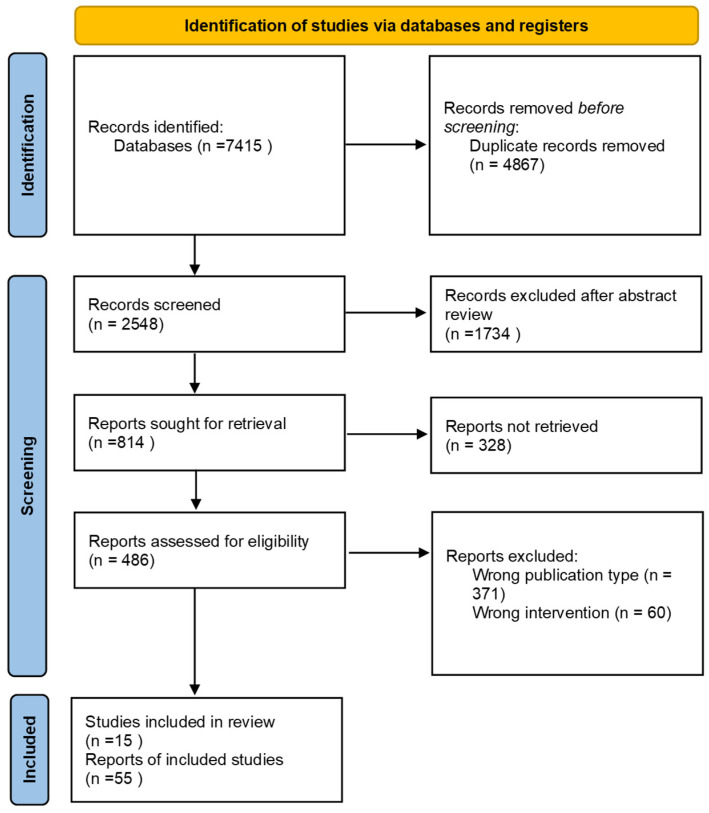
PRISMA flow-diagram of article selection process.

Literature research was conducted using databases such as PubMed and Google Scholar from 2008 onwards. The keywords used included: miRNA; breast cancer; liquid biopsy; cancer proliferation; cancer metastasis; diagnostic biomarkers; prognostic biomarkers; drug resistance; therapeutic targets. A total of 7415 publications were displayed. Search results were reviewed, putting emphasis on miRNA biogenesis and function in breast cancer, role of miRNA in breast cancer initiation, progression, metastasis and therapeutic resistance, diagnostic and prognostic potential of miRNAs in breast cancer, miRNA as liquid biopsy markers in breast cancer. Following the PRISMA guidelines, after exclusion of duplicates, abstract review for suitability and finally full-text analysis of remaining papers, a total number of 15 articles (Table 1) were selected for inclusion in the present review, as can be observed in the flow diagram (Figure 1).

This paper represents a scoping review and follows the reporting standards of the PRISMA 2020 statement. No formal protocol was registered for this review. Requirements regarding methodological transparency, data curation and study selection are stated in the Supplementary Materials.

## 3. Biology and Mechanism of miRNAs in Breast Cancer

### 3.1. miRNA Biogenesis and Function

MicroRNAs (miRNAs) represent an essential class of short, endogenous, non-coding RNAs, comprising between 18 and 25 nucleotides. These molecules serve as crucial regulators of post-transcriptional gene expression and exert their control by generally pairing with target messenger RNAs (mRNAs), leading to upcoming translational repression or degradation. The discovery of their ubiquitous presence and overregulation across mammalian organisms underscores their vital role in maintaining cellular homeostasis [15,28].

In oncology, especially in breast cancer development, the regulatory role of miRNAs is utterly important. Breast cancer is characterized by significant genetic and clinical heterogeneity. Breast malignancies are organized based on immunohistochemical markers such as Estrogen Receptor (ER), Progesterone Receptor (PR) and Human Epidermal Growth Factor Receptor 2 (HER2) status, encompassing categories such as Luminal A, Luminal B, HER2-positive, and Triple-Negative Breast Cancer (TNBC). Nevertheless, the molecular understanding of breast cancer progression indicates that dysregulation of the miRNA environment is an early and consequential event in the development of malignant tumors of the breast [11,28,29].

The unique pattern of miRNA expression observed in cancer is disease-specific and varies notably across molecular subtypes, suggesting that these signatures are very important determinants of clinical behavior and potential therapeutic vulnerability [28].

### 3.2. OncomiRs vs. Tumor-Suppressive miRNAs

MiRNAs are functionally classified based on their effect exerted on tumor development and progression, highlighting two main categories: Oncogenic MicroRNAs (Onco-miRs) and Tumor-Suppressor MicroRNAs (TS miRs). MiRNA expression is very often dysregulated in malignant tumors and the subsequent aberrant expression profile drives biological processes and changes such as proliferation, invasion, metastasis or apoptosis of malignant cells [30].

Onco-miRs are typically upregulated in malignant tumors and inhibit tumor-suppressor genes or pro-apoptotic pathways. Examples of such molecules frequently encountered in breast cancer include miR-21 and miR-155. Both onco-miRs are associated with unfavorable prognosis and elevated expression in aggressive breast malignancies subtypes such as Triple-Negative Breast Cancer (TNBC). MiR-21 exhibits an anti-apoptotic effect by targeting the 3′ untranslated region of PTEN (Phosphatase and Tensin Homolog) mRNA. Onco-miRs molecules are upregulated in breast cancer and promote carcinogenesis by repressing tumor suppressor genes. MiR-1297, miR-103b, and miR-498 are involved in leading breast carcinogenesis through direct targeting and repression of tumor suppressor gene PTEN. Furthermore, the oncogenic miR-200c-141 cluster mediates the metastatic potential of malignant breast cells by positively upregulating the expression of SerpinB2 [31,32,33].

Tumor-Suppressor MiRNAs are usually downregulated in cancer and inhibit oncogenes or pro-survival pathways. The miR-200 family, known to suppress malignant cell migration and invasion, is a fundamental exponent of Tumor-Suppressor MiRNAs category. The physiological function of Tumor-Suppressor MiRNAs is to inhibit cancer proliferation and progression by repressing the expression of oncogenes. Intense studied families, including let-7, miR-15/16, miR-34, and miR-200, exhibit strong tumor-suppressive activities focused on neutralizing oncogenic mRNA networks [31,34].

### 3.3. Role in Breast Cancer Initiation, Progression, Metastasis, and Resistance

Carcinogenesis in breast cancer is molecularly defined by the breakdown of mechanisms that enforce cellular growth control and discard damaged cells, primarily through the suppression of apoptosis. MiRNA molecules are involved in regulating essential cellular functions such as apoptosis, autophagy, or the epithelial–mesenchymal transition (EMT), therefore contributing to carcinogenesis [35].

Abnormal expression profiles of miRNAs can severely disrupt the mitochondrial pathway of apoptosis, which is a vital mechanism for triggering cell death in breast cancer. Oncogenic miRNAs actively work to undercut this process. Studies show that upregulated miRNAs, such as miR-27a, suppress apoptosis by supervising the BAK-SMAC/DIABLO-XIAP axis, a pathway that involves certain proteins (that activate mitochondrial permeabilization) and mediates cellular sensitivity to chemotherapy agents like Cisplatin. In a similar way, miR-32 promotes uncontrolled tumor cell proliferation while suppressing apoptosis through its target, FBXW7 [35].

Several miRNAs expressed during healthy breast tissue development are found to be dysregulated in breast cancer. This imbalance often involves a complete shift in function or expression level, leading to the re-activation of inherited (progenitor) programs, a usual characteristic of cancer initiation. For example, miR-205 is augmented in healthy progenitor cell populations, fulfilling the role of modulating cell differentiation. Expression level of miR-205 is observed to lessen significantly as breast cancer aggressiveness increases. This specific downregulation removes the healthy controls, allowing the malignant cell group to acquire a stem-like phenotype, promoting multiplication and invasiveness (metastasis). This finding confirms that tumor growth involves the systemic loss of key developmental controls mediated by miRNAs, demonstrating that studying miRNAs in the context of breast cancer development is vital for identifying mechanisms and targets for prompt treatment [36].

Tumor progression, mainly the processes of recurrence and resistance, is determined by the biology of Breast Cancer Stem-like Cells (CSCs), a small population possessing tumor-initiating properties. MiRNAs are decisive in regulating the “stemness” and pluripotency required to drive recurrence and progression of breast malignancy [37].

Invasion and metastasis represent the most aggressive characteristics of breast cancer, depending on the cell’s ability to transition into a translocating phenotype (epithelial–mesenchymal transition) and earn vascular support (process of angiogenesis) [35].

The regulatory influence of miRNAs on epithelial–mesenchymal transition is complex, controlling whether malignant cells remain confined to the primary site or acquire the necessary characteristics for invasion and systemic dissemination. The initial evidence linking miRNAs activity to the process of metastasis is centered on miR-10b. Overexpression of miR-10b in breast cancer cells that were otherwise non-invasive was sufficient to trigger tumor spread and distant metastasis in preclinical models [35,38].

The high rates of therapy resistance and tumor recurrence represent a major barrier to effective systemic treatment. MiRNAs are crucial mediators of acquired resistance across various therapeutic modalities, including chemotherapy, targeted and endocrine therapies (Table 2). The introduction of Trastuzumab improved the treatment of HER2-positive breast cancer. Nevertheless, a significant proportion of patients with HER2-positive breast cancer (up to 30%) still experience relapse after undergoing targeted therapy, often due to the acquisition of resistance mechanisms involving miRNAs. A critical example is the miR-221/PTEN axis. MiR-221 is observed to be upregulated in BC cells resistant to Trastuzumab therapy. This onco-miR promotes metastasis and resistance by directly targeting the tumor suppressor gene PTEN [18,34,39].

Endocrine therapies, such as Tamoxifen and Aromatase Inhibitors, are the foundation of treatment for estrogen-receptor positive breast tumors, but their effectiveness is reduced by acquired resistance in 40% to 50% of patients. MiRNAs are implicated as key disruptors that allow breast cancer progression even when estrogen signaling is chemically inhibited [40].

**Table 2:** Types of miRNAs involved in breast cancer therapeutic resistance.

Therapy	miRNA	Role in Resistance	Molecular Target or Pathway	Clinical Context
Trastuzumab ^1^ (Targeted)	miR-221 ^1^	Promotes resistance ^1^	PTEN ^1^	HER2-positive BC ^1^
Chemotherapy ^2^ (Docetaxel)	miR-21 ^2^	Provides resistance ^2^ (High expression)	Unknown targets leading to reduced susceptibility ^2^	MCF-7 BC cell lines ^2^
Chemotherapy ^2^ (Cisplatin)	miR-133 ^2^	Sensitiser ^2^ (Induction)	Increases cell sensitivity ^2^	Cisplatin-resistant TNBC cells ^2^
Chemotherapy ^2^ (Paclitaxel)	miR-155-5p ^2^	Overcomes resistance ^2^ (Transfection)	Unknown pathway modulation ^2^	Previously resistant BC cells ^2^
Endocrine Therapy ^3^	Various ^3^	Promotes progression and resistance ^3^	Disruptors of antiestrogen signaling pathways ^3^	Metastatic disease and acquired resistance ^3^

^1^—Ye et al., 2014 [18]; ^2^—Singh et al., 2023 [41]; ^3^—Muluhngwi et al., 2015 [40].

## 4. Diagnostic Potential of miRNAs: MiRNAs as Biomarkers in Breast Cancer

### 4.1. Circulating miRNAs as Non-Invasive Biomarkers (Liquid Biopsy)

The expression profiles of miRNAs are extremely dynamic across the various stages of the mammary gland life cycle, including adulthood, pregnancy, lactation, and involution. Studies involving murine models reveal hundreds of distinct miRNAs detected across these physiological stages (for instance 490 in adult stage and 419 in lactation phase). The miRNAs that are differentially expressed, nevertheless, often fall into expression clusters showing antagonistic profiles: certain miRNAs peak in adulthood and decrease during lactation, while others exhibit the opposite pattern [42].

An important observation highlighting the biological continuity between normal tissue regulation and malignant transformed tissue regulation is the identification of 32 miRNAs that are mutual expressed in the normal mammary tissue and during development of breast cancer. These shared regulatory molecules are involved in core cellular functions: proliferation, apoptosis, invasion and metastasis. This finding suggests that carcinogenesis is not driven by the introduction of entirely new regulatory elements, but rather by the dysregulation of a pre-existing controlling toolkit necessary for tissue development and recurrent remodeling. The mechanisms of breast cancer development often involve abnormal activation of certain pathways, reinforcing the need to study miRNA regulation in carcinogenesis, in order to inform therapeutic strategies [36].

Disruption of miRNA expression is a hallmark feature of breast cancer, affecting nearly all stages of neoplastic transformation. This disturbance results in functional categorization of miRNAs based on their final effect on tumor phenotype [16,43]. A critical aspect of miRNA biology is the potential for contradictory roles. Because a single miRNA can target several genes simultaneously, it is possible for a specific miRNA to suppress both oncogenic miRNAs and tumor suppressive miRNAs. Consequently, the conclusive effect—whether the miRNA acts as an onco-miR or as a TS-miR—is determined by the balance of expression levels of its multiple targets within the specific microenvironment of the tumor [36,44].

The chasing of minimally invasive diagnostic strategies for breast cancer has brought the concept of liquid biopsy to the forefront. This technique uses circulating miRNAs (c-miRNAs) found in body fluids (such as serum or plasma) as non-invasive biomarkers [45].

### 4.2. Tissue-Based miRNA Profiling

Breast cancer is highly heterogeneous, being classified into four distinct molecular subtypes: Luminal A, Luminal B, HER2-enriched and Triple-Negative Breast Cancer. The ability of miRNAs to differentiate these subtypes is crucial for customised diagnosis and treatment planning [13].

MiRNA profiling provides a potential standardized approach to classifying breast cancer subtypes, addressing current ambiguities in diagnostic practice. For instance, the process of differentiating the Luminal A subtype (which has a low proliferation index, Ki-67 < 20%) from the Luminal B subtype (with a high proliferation index, Ki-67 > 20%) can be influenced by inter-laboratory variability. The pioneering finding that miRNAs are dysregulated in early stages of ductal carcinoma development, showing distinct signatures between normal and malignant cells, highlighted essential molecules like miR-125b, miR-145, miR-21, and miR-155. Further research identified specific dysregulations, such as the downmodulation of miR-10b, miR-125b, and miR-145 and the upmodulation of miR-21 and miR-155, which correlate with pathological features like estrogen and progesterone receptor status [28,39].

The use of circulating miRNAs provides a promising non-invasive approach for diagnosis and prognosis, often referred to as a liquid biopsy. MiRNAs possess essential advantages for clinical use, notably their high stability within biological fluids like blood, plasma, or serum, which allows minimally invasive profiling and easier integration into routine procedures. This stability enables real-time and longitudinal observation of the disease course and monitoring of therapeutic response. Measuring miRNA expression in various biological substances or materials, including plasma and tissue, further increases their utility for both diagnostic and prognostic roles [46].

Tissue-based miRNA profiling implies analyzing miRNA expression directly within the tumor or adjacent tissue. MicroRNA microarrays can be used to identify panels of microRNAs that distinguish various types of malignant cells with greater sensitivity. The identification of breast cancer subtype-specific signatures (for instance the miR-99a/let-7c/miR-125b-2 cluster’s ability to differentiate Luminal A from Luminal B tumors) is a primary example of the diagnostic potential of tissue-based profiling. High-throughput sequencing has expanded this knowledge, identifying a number of 440 microRNAs associated with breast cancer, with 107 microRNAs indexed as potential biomarkers for detecting different types and stages of the malignant disease [39,46].

### 4.3. Common Diagnostic miRNAs in Breast Cancer

Consistently dysregulated miRNAs are now studied as key diagnostic markers, including the onco-miRNAs miR-21 and miR-155 and other clinically relevant types such as miR-10b, miR-195, miR-125b, and miR-145 [28,39].

MiRNA panels have demonstrated remarkable diagnostic potential, significantly surpassing the effectiveness of recognized protein biomarkers, such as CA15-3. The current recognized breast cancer biomarker, CA15-3, provides limited sensitivity (63.3%), specificity (60.7%), and an Area Under the Curve (AUC) of only 0.64 [46].

In contrast, a specific panel of circulating miRNAs, such as miR-4443, miR-572 and miR-150-5p achieved an AUC of 0.9366, demonstrating a sensitivity of 76.67% and a specificity of 97.22% in diagnosing breast malignancies. Furthermore, specific single miRNAs, such as miR-133a-3p (AUC 0.84), have shown a strong ability to distinguish breast cancer from benign breast tumors. This superior performance provides compelling evidence for the potential of miRNAs to improve early detection, especially in asymptomatic stages or in patients with negative CA15-3 results [46]. Furthermore, miRNAs offer a significant diagnostic advantage for patients with equivocal or non-specific imaging findings. In cases where traditional mammography or ultrasound results are inconclusive, these molecular biomarkers can provide a non-invasive ‘liquid biopsy’ to help differentiate between benign and malignant lesions. By identifying specific miRNA signatures associated with early oncogenesis, clinicians may be able to achieve a definitive diagnosis earlier.

## 5. Prognostic Value of miRNAs

### 5.1. miRNAs Correlated with Tumor Grade, Subtype, and Survival Outcomes

MiRNA expression profiles are often dysregulated, leading to their categorization based on function: oncogenic miRNAs and tumor-suppressor miRNAs. Onco-miRNAs, such as miR-21 and miR-155, are typically overexpressed in malignant tissues and contribute to tumor progression by inhibiting the expression of vital tumor suppressor genes. Conversely, tumor-suppressor miRNAs, exemplified by miR-143, miR-145, and miR-34a, are markedly downregulated in cancer, and the loss of their function facilitates aggressive cancer phenotypes. This dual role, coupled with an overall downregulation of miRNA expression compared to normal tissues, sets up miRNAs as fundamental components of carcinogenesis [43,47,48,49].

MiRNAs play a significant role in stratifying breast cancer patients based on molecular subtypes and long-term risk. MiR-21, a common onco-miRNA, exhibits consistent overexpression correlated with advanced tumor stage, lymph node metastasis and significantly diminished patient survival. Univariate Cox analysis identified high miR-21 expression as the most important predictive factor for poor prognosis in breast cancer patients, outweighing the prognostic impact of clinical stage and histological grade. In quantitative terms, the 5-year survival rate for patients with high miR-21 expression was 45.90%, notably lower than the 86.54% rate observed in patients with low expression [17,43].

The prognostic value of miR-21 is highly dependent on disease stage. In the early-stage of cancer development, high miR-21 expression was correlated significantly with poor survival. However, within the late-stage group, miR-21 expression showed no statistically significant relationship with patient survival, suggesting that its strongest clinical utility lies in identifying high-risk individuals early in the disease course. Beyond miR-21, other markers such as miR-9, are associated with hormone receptor status (ER status). Combining miRNA profiles (e.g., miR-155, miR-24) with established protein markers like Ki-67 can increase the prediction of relapse risk [17,43].

### 5.2. Predicting Response to Therapy (Chemo-, Endocrine, Targeted Therapies)

MiRNA function is not only to anticipate general outcomes but also to predict the specific effectiveness of distinct therapeutic modalities. Their ability to reflect and drive resistance mechanisms makes them indispensable tools for customed oncologic treatment [14,40].

MiRNAs are disruptors of endocrine therapies (antiestrogen agents) by regulating pathways that confer resistance, making them subjects of keen interest in metastatic breast cancer. Specific exosomal miRNAs (miR-221 and miR-222) have been shown to contribute to Tamoxifen resistance in Estrogen Receptor (ER)-positive breast cancer cells by targeting SFRP1 (secreted frizzled-related protein 1), by that means promoting tumor progression [14,40].

In HER2-positive breast cancer, exosomal miR-1246, either individually or in combination with miR-155, can predict Trastuzumab resistance by promoting malignant cell proliferation and invasion [50].

### 5.3. Role in Monitoring Recurrence or Metastasis

The application of microRNAs in cancer surveillance addresses a critical clinical need for non-invasive, repeatable, and sensitive monitoring of disease activity, especially recurrence and metastasis. Circulating and exosomal miRNAs are uniquely suited for this role due to their accessibility and reflection of dynamic tumor biology [51].

MiR-21 is recognized as one of the most significant and widely upregulated onco-miRNAs, involved in the process of carcinogenesis across various types of cancer, including breast, lung, ovarian, and colorectal malignancies. Its dominant oncogenic action is executed through the targeting and subsequent downregulation of master tumor suppressor genes, most notably PTEN (Phosphatase and Tensin Homolog). The suppression of PTEN gene leads to the uncontrolled activation of the pro-survival signaling pathways, including the AKT and Ras/MAPK pathways, which collectively drive increased cell proliferation, migration, and invasion [52,53,54].

## 6. Classification and Types of miRNAs in Breast Cancer

### 6.1. Oncogenic vs. Tumor-Suppressor miRNAs

OncomiRs represent microRNAs that promote the development and progression of malignancy. They achieve their oncogenic potential by targeting and destabilizing the mRNAs of tumor suppressor genes or by inducing pathways that encourage cell survival and cancer proliferation [55].

Tumor-suppressor microRNAs (TS-miRNAs) play a defensive role by modulating or suppressing the expression of oncogene-coding genes, therefore preventing carcinogenesis and tumor progression. The downregulation of these TS-miRNAs can be seen in many types of cancer and restoring their expression often provides an effective anti-tumor phenotype [56].

### 6.2. Intracellular vs. Extracellular (Circulating/Exosomal) miRNAs

MiRNAs can be organised based on their location: intracellular (within the primary tumor or target cells) or extracellular (circulating in body fluids). Intracellular miRNAs exert primary regulatory functions within the malignant cells of the primary tumor. Their identification and quantification require direct tissue sampling. Standard quantification methods include Reverse Transcription quantitative PCR (RT-qPCR), which is highly sensitive, but restricted to the detection of known miRNAs. A more comprehensive, but more expensive and time-consuming, approach is Next Generation Sequencing (NGS), which allows the detection of numerous miRNAs, including novel ones, with high sensitivity [43,51].

Circulating miRNAs are highly stable and easily detectable in many biological fluids, including peripheral blood, urine, plasma or saliva. This remarkable stability, which is essential for clinical utility, is achieved by packaging the miRNAs within protective carriers, most notably Extracellular Vesicles (EVs), such as exosomes [57,58].

### 6.3. Panels vs. Single-miRNA Approaches

While certain single miRNAs, such as miR-21 and miR-155, reveal significant roles in cancer progression and are consistently upregulated in patient samples, their individual diagnostic performance can be limited for routine clinical use due to issues related to specificity and the complexity of disease biology. However, even the total circulating level of miRNAs can be used as an independent prognostic marker for risk assessment [13].

The association of multiple miRNA biomarkers into a panel significantly improves diagnostic accuracy and clinical prognosis. This enhanced performance is revolutionary in the field of non-invasive screening methods [13].

## 7. miRNA Detection and Quantification in Liquid Biopsies

Liquid biopsy samples for circulating miRNA analysis can be obtained from a variety of body fluids, including blood (serum or plasma), urine, saliva, and even breast-specific fluids such as nipple aspirate fluid or breast milk [59,60]. Among these, blood is the most widely used source in breast cancer studies, given its abundance of tumor-derived exosomal miRNAs and ease of repetitive collection [13]. Other biofluids are under investigation; for example, urinary miRNAs have shown promise as non-invasive breast cancer biomarkers, and salivary miRNAs are being explored for cancer diagnostics in general [60]. Regardless of source, miRNAs in biofluids are often protected from RNase degradation by being enclosed in extracellular vesicles or bound to proteins like Argonaute-2 or high-density lipoproteins [46].

Extraction and purification of miRNA from liquid biopsies typically involve isolating total RNA (enriched for small RNAs) using methods adapted to low concentrations. Two common approaches are organic extraction (phenol–chloroform based) and silica column-based kits. Organic phase separation (e.g., with TRIzol or similar reagents) is widely used and can yield high RNA amounts, but it involves toxic chemicals and multiple steps [61]. Spin column kits (e.g., Qiagen miRNeasy) offer a simpler, safer workflow, binding small RNAs to a silica membrane for purification. Newer magnetic bead-based protocols allow automated, high-throughput extraction of circulating miRNAs [62]. Choice of method can impact yield and miRNA profiles: studies comparing kits have found significant variability in recovered miRNA quantity and quality, underscoring the need for protocol standardization. To improve consistency, addition of carrier molecules and rigorous control of pre-analytical variables (like avoiding hemolysis during blood draw) are recommended [61,62,63].

After RNA isolation, miRNA detection and quantification rely on sensitive molecular techniques. The gold standard for measuring known miRNAs is reverse transcription quantitative PCR (RT-qPCR) [20,22]. RT-qPCR assays (often using stem-loop RT primers and TaqMan or SYBR Green chemistry) can reliably quantify specific miRNAs from plasma or other fluids with high sensitivity, even when starting RNA is limited [20]. For profiling multiple miRNAs simultaneously, microarray-based hybridization was historically used, though it has largely been superseded by next-generation sequencing (NGS) for discovery purposes. Small RNA sequencing (RNA-seq) allows an unbiased, genome-wide identification of miRNAs in biofluids and can detect novel or unanticipated miRNA species. However, NGS requires deeper sequencing to capture low-abundance miRNAs and involves complex data analysis. An emerging alternative for highly sensitive quantification is digital PCR (such as droplet digital PCR), which can provide absolute miRNA copy numbers and improved precision for low-level targets. In addition, innovative detection methods are being developed, including isothermal amplification strategies, electrochemical biosensors, and nanoparticle-based assays to directly detect circulating miRNAs [19,64].

Detecting miRNAs in liquid biopsies comes with technical challenges and limitations. One major challenge is the low abundance of many circulating miRNAs, which necessitates PCR amplification or other signal enhancement steps to reach detectable levels. The minute quantities also make results susceptible to contamination or variation from sample handling. Lack of standardized normalization is another concern—unlike tissue mRNA assays, there is no universally accepted endogenous control for circulating miRNAs. Researchers often use exogenous spiked controls or normalize to a subset of stably expressed miRNAs, but different studies employ different strategies, complicating comparisons. Furthermore, disparities in protocols (from sample collection to RNA extraction and assay platform) have led to inconsistent findings across studies. For instance, a multi-center analysis noted that different extraction kits and analysis platforms can yield non-overlapping miRNA profiles from the same samples, emphasizing the need for cross-study standardization. Pre-analytical variables also critically affect detection: delays in processing blood can lead to RNA degradation or release of background miRNAs from blood cells, and even slight hemolysis can artificially elevate certain miRNAs abundant in erythrocytes or platelets. These factors demand careful control—e.g., using dedicated blood collection tubes, immediate plasma separation, and consistent storage conditions—to ensure that measured miRNA differences reflect biological signal rather than technical artifact [20,22,62,63].

## 8. MiRNAs as Liquid Biopsy Markers in Breast Cancer

Extensive research in the past decade has demonstrated that circulating miRNAs can serve as informative biomarkers for breast cancer. Numerous clinical studies have profiled miRNA expression in blood samples from breast cancer patients and healthy controls, consistently finding dysregulated miRNAs associated with the presence of cancer. For example, oncomiRs such as miR-21 and miR-155 are frequently elevated in the plasma of breast cancer patients and were among the first candidates identified as circulating biomarkers. These miRNAs, known to be upregulated in breast tumor tissue, are also released into circulation, reflecting the tumor’s molecular signals. Indeed, certain circulating miRNA patterns mirror those found in the tumor itself: Hannafon et al. reported that miR-21 and miR-1246 are present at remarkably high levels in exosomes from the plasma of breast cancer patients, consistent with their overexpression in tumor cells. Such concordance supports the idea that liquid biopsy miRNA profiles can partly represent the molecular makeup of the cancer. That said, not all circulating miRNAs originate exclusively from tumor cells—some may come from the surrounding microenvironment or immune response—but in aggregate they provide a tumor “fingerprint” in the bloodstream [20,22,23,59,65].

Multiple comparative studies have directly examined the miRNA profiles of liquid biopsies versus tissue biopsies. Generally, there is overlap in key oncogenic miRNAs but also differences: circulation may preferentially contain miRNAs from aggressive tumor subclones or from metastatic sites that shed vesicles into blood. For instance, miR-10b and miR-34a might be detectable in primary tumor tissue but their levels in blood could depend on active release mechanisms. Despite these nuances, a recurring observation is that panels of circulating miRNAs can distinguish breast cancer patients from disease-free individuals with high accuracy. A recent study developed and validated a blood-based panel of four miRNAs (miR-24, miR-206, miR-373, and miR-1246) for early breast cancer detection, achieving an area under the ROC curve (AUC) ~0.97 with 98% sensitivity and 96% specificity in one cohort. Another group identified a three-miRNA plasma signature that yielded ~92% sensitivity and 90% specificity for breast cancer in a case–control study. These high-performing signatures underscore the potential of circulating miRNAs as non-invasive diagnostics. Importantly, some circulating miRNA markers have shown efficacy even for early-stage tumors: for example, Heneghan et al. reported that serum miR-195 could detect early breast cancer and differentiate it from other malignancies, highlighting its possible use in screening [19,20,21,24,27].

Beyond diagnosis, circulating miRNAs hold value for prognosis and disease monitoring. Certain miRNA levels in blood correlate with tumor burden, stage, or aggressiveness. For example, elevated plasma miR-155 has been associated with more advanced disease and poorer survival outcomes in breast cancer, and a meta-analysis indicated that circulating miR-155 can facilitate accurate detection of breast cancer across studies (pooled diagnostic AUC ~0.92). Differences in miRNA profiles have also been observed between breast cancer subtypes—for example, unique miRNA signatures are reported in triple-negative vs. luminal tumors—suggesting that liquid biopsy miRNAs might help non-invasively subclassify cancer biology. Indeed, one study found that a combination of two circulating miRNAs (miR-142-5p and miR-320a) could distinguish luminal A subtype patients from healthy individuals with high specificity (AUC 0.94) in plasma. Moreover, comparisons of miRNA content in exosomes versus whole plasma have revealed that exosomal fractions may enrich for tumor-specific miRNAs, potentially increasing the signal-to-noise ratio for biomarker detection. For example, isolating serum exosomes and measuring miR-1246 significantly improved diagnostic accuracy for early breast cancer in one report, compared to measuring miR-1246 in unfractionated plasma [22,23,65,66].

The clinical utility of miRNA-based liquid biopsy tests is being actively evaluated. One attractive application is early detection of breast cancer in high-risk or general populations using a blood test. While no miRNA test is yet approved for routine screening, the aforementioned multi-miRNA panels demonstrate that this approach is technically feasible, and ongoing trials are assessing whether adding circulating miRNA assays could complement mammography for earlier diagnosis. Circulating miRNAs also show promise in monitoring disease progression and recurrence. Because miRNA levels can dynamically reflect tumor dynamics, serial blood tests might detect rising levels of specific miRNAs that herald recurrence or metastasis before imaging does. For instance, in metastatic breast cancer patients, changes in circulating miR-10b and miR-373 have been noted to correlate with disease progression on therapy. A 2022 study reported that the plasma levels of certain miR-200 family members tracked tumor response in metastatic disease: patients who responded to systemic therapy showed decreasing miR-200c/miR-141 levels, whereas increases portended treatment resistance. Such findings suggest a role for miRNAs in real-time monitoring—a liquid biopsy drawn during therapy could indicate whether a tumor is responding or resistant, enabling earlier changes in treatment. Additionally, circulating miRNAs have been investigated as predictive markers of treatment response. One notable example is a serum miRNA signature that predicts benefit from trastuzumab in HER2-positive breast cancer: a study identified that patients with a specific four-miRNA profile had significantly better responses to trastuzumab, whereas those lacking it derived minimal benefit. This kind of blood test could guide personalized therapy decisions, sparing certain patients from ineffective treatments. Similarly, plasma miR-21 has been proposed as a marker of chemosensitivity—Anwar et al. showed that rising circulating miR-21 levels were associated with poor response to chemotherapy and shorter survival, indicating it could be used to identify non-responders early. Furthermore, a pilot study of exosomal miRNAs found distinct expression patterns in patients who achieved pathologic complete response to neoadjuvant chemotherapy versus those who did not, implying a predictive role for exosomal miR-34a, miR-16 and others in neoadjuvant settings. Although these applications require further validation, they illustrate the versatile clinical applications of miRNA-based liquid biopsies—from early detection and molecular diagnostics to prognostication and therapeutic monitoring in breast cancer [19,24,25,26,27,67].

## 9. Advantages of miRNAs as Biomarkers

MicroRNAs offer several compelling advantages as cancer biomarkers, especially in the context of liquid biopsies. First, they enable non-invasive testing. Unlike tissue biopsies that require surgery or needle extraction, circulating miRNAs can be measured from blood draws or other body fluids, minimizing discomfort and risk to patients. This non-invasiveness not only makes repeat sampling feasible but also allows biomarker testing in patients who may not be candidates for frequent biopsies. The ability to perform serial liquid biopsies is invaluable for ongoing monitoring and early detection of changes. In breast cancer management, a blood-based miRNA test could be done regularly during follow-up, complementing imaging by potentially catching molecular signs of relapse earlier. Thus, miRNA biomarkers fulfill the desire for minimally invasive yet informative diagnostics [12,66].

Secondly, circulating miRNAs have remarkable stability in body fluids. Despite the RNase-rich environment of blood and other fluids, miRNAs are protected from degradation by their packaging. They are secreted in membrane-bound exosomes or microvesicles or bound to stable protein complexes, which shield them from enzymatic breakdown. This intrinsic stability means that miRNAs remain intact under typical clinical sample handling—for example, studies have shown that miRNAs are not significantly degraded by multiple freeze–thaw cycles or prolonged storage in biofluids (within reasonable limits). In contrast, other biomarkers like mRNA are far more fragile in circulation. The high stability of miRNAs enables reliable detection and quantification. It also implies that archival serum/plasma samples can be analyzed for miRNAs, facilitating retrospective studies. In short, miRNAs’ resistance to degradation is a key practical advantage for developing robust blood tests [63].

Another advantage is the disease specificity and dynamic expression of many miRNAs. MiRNA expression is tissue-specific and dysregulated in a disease-specific manner—certain miRNAs are characteristically elevated in breast cancer but not in other conditions, helping to distinguish patients with malignancy. For example, miR-155 and miR-21 are commonly associated with breast cancer, whereas other circulating miRNAs might be indicative of different cancers or benign diseases. By analyzing a panel of multiple miRNAs, tests can achieve high specificity for breast cancer (as opposed to a general inflammatory state). Moreover, miRNA levels change dynamically with disease state: they tend to rise with increasing tumor burden or aggressive biology and may fall after successful treatment. This dynamic range allows miRNAs to function as real-time indicators of tumor behavior. In contrast, static markers (like germline DNA mutations) do not vary with disease activity. The fact that miRNAs are actively released by cancer cells means they can provide a contemporaneous readout of what the tumor is doing, making them ideal for real-time monitoring. For example, within weeks of starting therapy, decreases in certain oncogenic miRNAs can signal treatment efficacy, whereas rebounds in those miRNAs might precede radiographic progression [22,26,66,67].

MiRNA biomarkers are also complementary to imaging and histopathology. Traditional imaging like mammography, MRI, or CT provides anatomical information but can miss microscopic disease and often cannot be repeated too frequently due to cost or radiation exposure. Histopathology from biopsies gives definitive tissue diagnosis but is invasive and typically only samples a single tumor site. Circulating miRNAs, on the other hand, can capture molecular information from multiple tumor foci (including metastases) that shed nucleic acids into the circulation [65]. They can reflect changes that are not yet visible on scans, such as molecular recurrence before a tumor becomes large enough to detect. As an adjunct to imaging, a rise in a specific miRNA could prompt closer examination or earlier intervention even when scans are equivocal. Likewise, miRNAs can add quantitative molecular data to complement tissue biopsy findings—for example, a low-risk pathology might be re-evaluated if a blood miRNA signature suggests high-risk disease biology. In essence, miRNA assays provide a different layer of information (molecular and systemic) that augments the local and structural insights from imaging and biopsy [65].

Crucially, circulating miRNAs hold potential for early detection of cancer. Because tumors may release miRNAs into circulation at very early stages, a sensitive miRNA-based test could detect cancers when they are still small or clinically imperceptible. Indeed, some miRNAs have been found elevated in the blood of patients with early-stage (I/II) breast cancer compared to cancer-free controls (including miR-195, miR-21, and others). In principle, a panel of such miRNAs could serve as a screening tool to catch breast cancer before it progresses, potentially improving survival through earlier intervention. The high stability and detectability of miRNAs in routinely collected fluids (like blood or even potentially breast milk for postpartum women support their use in population screening, if sufficient sensitivity and specificity can be attained. Even incremental improvements in early detection rates via miRNA assays could translate into significant mortality reductions, given breast cancer’s outcomes are strongly stage-dependent [20,21,59].

Lastly, miRNA profiles can enable personalized medicine applications. Each patient’s tumor may have a unique miRNA signature that not only aids in diagnosis but also provides prognostic and predictive information. By decoding this “miRNA fingerprint,” clinicians could tailor management strategies to the individual [25]. For example, a patient whose plasma harbors a high-risk miRNA signature (associated with poor outcomes) might benefit from more aggressive therapy or intensive monitoring, whereas a patient with a more “benign” miRNA profile could be a candidate for de-escalation of treatment. Moreover, specific miRNAs can indicate vulnerabilities in the tumor. If a tumor releases high levels of miRNAs linked to a certain pathway (say, PI3K/AKT activation), targeted inhibitors for that pathway might be particularly effective for that patient. In this way, circulating miRNAs act as both biomarkers and surrogates for the tumor’s genetic/epigenetic landscape, guiding targeted treatment decisions. A concrete example is the earlier-mentioned miRNA signature predicting trastuzumab benefit in HER2+ breast cancer: using that signature, one could personalize therapy by identifying which patients should indeed receive trastuzumab versus those who might need an alternative approach. As more is learned about miRNA functions, there is also interest in therapeutically targeting miRNAs (using miRNA mimics or inhibitors), which could open the door to miRNA-based therapies. In summary, the non-invasive nature, stability, specificity, and real-time responsiveness of circulating miRNAs, along with their reflection of underlying tumor biology, make them exceptionally attractive as biomarkers in breast cancer [25].

## 10. Limitations and Challenges

Despite their promise, there are significant limitations and challenges that must be addressed before circulating miRNAs can be fully integrated into clinical practice. One set of challenges revolves around pre-analytical variables and standardization. The process of obtaining and handling liquid biopsy samples can introduce variability in miRNA measurements. Factors such as the blood draw technique, type of collection tubes, time to plasma separation, and storage conditions (temperature, duration, freeze–thaw cycles) can all influence miRNA levels. As an example, hemolysis (rupture of blood cells during phlebotomy or processing) can release intracellular miRNAs (like miR-16 or miR-451 from red blood cells) into the plasma, confounding the assay results. Without strict protocols, two laboratories might obtain different miRNA profiles from the same patient simply due to differences in pre-analytical handling. This lack of standardization has been a major hurdle in reproducing biomarker findings across studies. Therefore, standard operating procedures for sample collection and processing need to be established. Efforts are underway—for example, consortium guidelines suggest using EDTA tubes and processing blood within hours of collection, and quantifying hemolysis by spectrophotometry to exclude compromised samples. Additionally, differences in miRNA extraction and quantification methods between studies have led to inconsistent results. As noted, there is no single accepted method to measure circulating miRNAs; some studies use qPCR with one technology, others use sequencing or arrays, each with its own biases. A given miRNA might appear highly abundant with one platform and moderate with another due to technical factors. Until the field agrees on validated, standardized assays (analogous to how blood glucose is measured by a standard enzymatic method in all labs), it will be challenging to compare results and set universal cut-offs. The lack of standardized quantification and normalization methods is thus a critical challenge. Researchers are exploring the use of spike-in control miRNAs or consensus reference miRNAs to normalize data, but no clear consensus has emerged. This makes regulatory approval difficult, since regulators require robust reproducibility. In summary, controlling pre-analytical variables and standardizing methodologies are top priorities to ensure that miRNA biomarker tests are reliable and transferable to clinical settings [20,61,62,63].

Another challenge is biological variability in circulating miRNA levels. MiRNA profiles can be influenced by a person’s age, hormonal status, ethnicity, comorbid conditions, or even acute stress and inflammation. In women, factors like the menstrual cycle or pregnancy can alter the circulating levels of certain miRNAs (for example, placental microRNAs can be detected during pregnancy). Such physiological variations can complicate the interpretation of a positive biomarker result—a mild elevation in a particular miRNA might be due to a non-cancer reason in some individuals. Large studies will be needed to define baseline distributions of key miRNAs in healthy populations, accounting for these variables, to set appropriate thresholds that maximize cancer specificity. Additionally, tumor heterogeneity poses a limitation. Breast cancer is a heterogeneous disease; different subtypes (luminal vs. HER2+ vs. triple-negative) and even different clones within the same tumor can have distinct miRNA expression patterns. A single circulating miRNA or small panel may not capture all cases—some tumors might not release the specific miRNAs being measured. For example, a miRNA panel derived from mostly HER2+ patient data might underperform in triple-negative patients if their tumors use a different set of miRNAs. Heterogeneity also extends to metastases: a patient with metastases in the liver and bone might shed a broader mix of miRNAs than a patient with only an in situ tumor. This diversity means that any universal miRNA biomarker test for “breast cancer” might miss a fraction of cases or need subtype-specific adjustments. It may be necessary to develop subtype-tailored biomarker panels or broad combinations to cover the spectrum of disease. Tumor heterogeneity also implies that correlations between blood miRNA levels and actual tumor burden can vary—in some cases a small but biologically active tumor could secrete disproportionate amounts of a miRNA, whereas a large tumor of a subtype that releases little miRNA could evade detection. Managing these biological differences is a challenge for sensitivity and generalizability of miRNA tests [23,62,63].

A further limitation is the current lack of large-scale validation. Many circulating miRNA biomarkers have been identified in relatively small, single-center studies or retrospective cohorts. Few have been tested in prospective trials or across multiple centers and diverse populations. This raises concerns about overfitting and false-positive results in the literature. Some early reported biomarkers did not hold up when re-evaluated in larger cohorts or by independent groups. To become clinically reliable, candidate miRNA signatures must be validated in robust, blinded studies (ideally, prospective collection with predefined endpoints). For instance, a miRNA-based diagnostic panel would need to be trialed in a screening population to see if it indeed catches cancers and reduces false positives at an acceptable rate. Likewise, a prognostic or predictive miRNA marker should be confirmed in multi-center studies to ensure it works broadly. Until such validation is done, these biomarkers remain exploratory. The regulatory and implementation challenges are non-trivial as well. Even if a miRNA test shows promise, it needs to gain regulatory approval (e.g., FDA approval) which demands demonstration of clinical validity and utility. This process is lengthy and requires consistent assay performance. As of 2025, no circulating miRNA test for breast cancer has been approved for routine clinical use, reflecting the gap between research findings and clinical translation. Moreover, clinicians would need education and guidelines on how to use miRNA test results in decision-making. Integration into clinical workflows (when to order the test, how to act on a positive or negative result) has to be established. There may also be economic considerations—the cost of specialized miRNA assays (especially if NGS-based) could be high, and payers will want evidence of cost-effectiveness (for example, does a miRNA test prevent enough advanced cancers or unnecessary biopsies to justify its expense?) [12,19,20,66].

Another technical challenge is that miRNAs are present at low concentrations, often near the limits of detection of assays. This means assays must be extremely sensitive and careful to avoid analytical false negatives. Low abundance also accentuates any background noise: a slight contamination or assay variability can have a large relative effect. Achieving a low limit of detection with high precision is therefore essential and not all labs may have the required infrastructure or expertise initially. Lastly, there are challenges in data interpretation—unlike single-gene mutations, miRNA signatures often involve multiple markers whose combined pattern determines the result. This can be less intuitive for clinicians, and robust computational models are needed to interpret results (e.g., a machine learning classifier that outputs a risk score from a 5-miRNA panel). Ensuring these models are transparent and validated is important to gain clinical trust [22,68].

In summary, while circulating miRNAs are promising, one must contend with pre-analytical and analytical standardization issues, biological variability and tumor heterogeneity, a paucity of large validation studies, and hurdles in clinical implementation. Addressing these challenges—through improved protocols, collaborative studies, and technology development—will be crucial for the successful translation of miRNA biomarkers into reliable tools for breast cancer management [68].

## 11. Future Directions

The field of miRNA biomarkers in breast cancer is rapidly evolving, and several future directions are likely to enhance their diagnostic and prognostic utility. One key avenue is the integration of miRNA analysis with multi-omic approaches. Rather than evaluating miRNAs in isolation, researchers are beginning to combine miRNA profiles with other liquid biopsy components—such as circulating tumor DNA (ctDNA), circulating proteins, or metabolites—to create multi-analyte blood tests. Multi-omic signatures could leverage the strengths of each biomarker type: for example, a panel might incorporate ctDNA mutations for tumor specificity, protein markers for context, and miRNAs for early dynamic changes. In breast cancer, a combined assay of ctDNA and miRNAs might improve sensitivity for early detection, as some tumors that shed insufficient DNA might still release detectable miRNAs and vice versa. There is also interest in pairing miRNA data with imaging (radiogenomics)—for instance, whether a certain miRNA signature plus a suspicious imaging finding yields a higher predictive value for malignancy than imaging alone. As computational methods advance, integrating these diverse data types may uncover robust composite biomarkers. A proof-of-concept study showed that combining plasma miRNA levels with conventional protein tumor markers improved diagnostic accuracy in differentiating benign from malignant breast lesions. In the coming years, large-scale projects (akin to The Cancer Genome Atlas, but for liquid biopsy) may profile thousands of patients across multiple “omes” to discover the best combination of markers for detection and monitoring [65,66,69].

Another future direction is the application of artificial intelligence (AI) and machine learning to miRNA biomarker analysis. Machine learning algorithms are well-suited to detect subtle patterns in high-dimensional data, which describes miRNA profiles. To date, studies have employed algorithms like support vector machines or random forests to build classifiers that distinguish cancer vs. non-cancer based on dozens of circulating miRNAs. As datasets grow, more sophisticated AI (including deep learning) could be used to identify complex miRNA signatures that humans might not discern. These models can also incorporate clinical data (age and risk factors) alongside miRNA values to increase predictive power. A machine learning-based 3-miRNA classifier for breast cancer diagnosis has shown excellent performance in a controlled study, and similar approaches could be trained on detecting recurrence or predicting therapy response. In the future, we might see AI-driven decision support where an algorithm analyzes a patient’s blood miRNA spectrum and outputs a likelihood of having a malignancy or a likely tumor subtype. However, to implement this clinically, large training datasets and external validations will be needed to ensure that models are accurate and generalizable. Additionally, AI can aid in identifying novel biomarker candidates by analyzing which miRNAs (or combinations) best stratify outcomes in retrospective data. In essence, machine learning will be a powerful tool to fully exploit the informational richness of circulating miRNAs and to handle the complexity of multi-marker assays [20,22,25,65,68].

Advances in personalized medicine will also shape the future of miRNA biomarker applications. We expect a move towards tailoring not only treatments but also surveillance strategies based on miRNA profiles. For example, a patient with a circulating miRNA signature indicative of minimal residual disease after surgery might receive closer monitoring or adjuvant therapy intensification, whereas another patient with no adverse miRNA signals could be monitored less aggressively. Furthermore, as the mechanistic roles of specific miRNAs in driving breast cancer behavior are elucidated, these miRNAs might become targets or tools for therapy. Some ongoing clinical trials are already exploring miRNA-based therapeutics (e.g., miR-21 inhibitors or let-7 mimics in cancer therapy)—positive results could reinforce measuring those miRNAs as both a way to select patients and to track therapeutic effect. In terms of targeted treatment decisions, one could envision, for instance, using circulating miR-34a levels to decide on using a p53 pathway reactivator, if research finds that low miR-34a correlates with p53 dysfunction. The information from miRNA patterns might also guide combination therapies by highlighting multiple pathways active in a patient’s tumor. The ultimate vision of precision oncology is that each patient’s disease is profiled (including by liquid biopsy miRNAs) and therapies are customized accordingly; the continued integration of miRNA data into this paradigm is expected [25,27,65,66].

Large-scale prospective validation studies are on the horizon to pave the way for clinical implementation. International consortia and trials are needed to test circulating miRNA biomarkers in real-world settings. For example, a prospective screening trial could evaluate whether adding a miRNA blood test for high-risk women leads to detection of cancers that were missed by imaging and whether this improves outcomes. Similarly, a prospective trial in the adjuvant setting could investigate if patients with a positive post-surgery miRNA marker (suggesting residual disease) benefit from additional therapy compared to marker-negative patients. Such studies will provide the evidence of clinical utility required by regulatory bodies and guideline committees. They will also help determine practical aspects like optimal sampling frequency (how often to test) and turnaround time. We anticipate that within the next 5–10 years, results from these studies will clarify which miRNA biomarkers truly add value to standard care [19,20,66,68].

Finally, efforts toward standardization and clinical translation frameworks will be crucial future steps. Stakeholders are likely to develop consensus guidelines for circulating miRNA assay performance, analogous to existing guidelines for PCR-based tests (e.g., the MIQE guidelines for qPCR). These might cover recommended sample handling, validation procedures, and quality control metrics. Regulatory science will also catch up: agencies may draft specific frameworks for evaluating diagnostic miRNA signatures, including requirements for reproducibility and perhaps even bioinformatics components (since many assays will involve computational classification). The establishment of reference materials—such as plasma samples with known miRNA concentrations or synthetic miRNA standards—could facilitate proficiency testing and calibration across laboratories. On the clinical side, incorporating miRNA tests into diagnostic pathways will require clear algorithms; for example, how to act on a positive screening result (biopsy immediately or repeat test?), or how to modify therapy based on a monitoring result. As a step in this direction, some companies and academic groups are already developing kit-based miRNA tests for research use, which could streamline adoption if approved clinically. Collaboration between oncologists, laboratorians, and data scientists will be essential to smoothly integrate these new tests. In summary, the future will likely bring more integrated biomarker strategies, powered by advanced analytics and validated by large trials, that elevate circulating miRNAs from a research tool to a routine component of breast cancer care. The field is moving toward making the liquid biopsy—with miRNAs as a key element—an established part of precision oncology [12,63,68,70].

## 12. Conclusions

Exploring the role and importance of miRNAs in the context of breast cancer is still a challenging task, but one of great importance. MicroRNAs provide significant advantages as cancer biomarkers, particularly for their utility in liquid biopsy applications. The path to clinical implementation relies on upcoming large-scale validation studies. To ensure these miRNA biomarkers work in everyday medical practice, we must establish international consortia and multi-center trials. While circulating miRNAs hold great promise, substantial challenges—particularly regarding pre-analytical variables and standardization—must be resolved before clinical integration is feasible. Nevertheless, the rapid evolution of miRNA research in breast cancer suggests that emerging methodologies will soon improve their diagnostic and prognostic accuracy.

## Figures and Tables

**Table 1:** Characteristics of studies included in the analysis.

	Author	Year	Number of Subjects/Samples	Type of miRNA Analyzed	Aim of Research	Findings
1.	Agrawal P [14]	2025	Pan-cancer data (specific number not in citation)	Exosomal miRNAs	To characterize the pan-cancer role of exosomal miRNAs in metastasis across different cancer types	Emphasized the bidirectional interaction between the gut microbiome and host miRNAs
2.	Degheidy MS [11]	2025	N/A	miR-155-5p, miR-21-5p, miR-93-5p, miR-140-5p	To investigate the regulatory roles of these four miRNAs in breast cancer progression	miRNA-133a-3p and miRNA-21 are highly reliable indicators for predicting mortality and progression in inflammatory states
3.	Djuranovic S [15]	2012	N/A (Fundamental cell line study)	N/A	To define the mechanism of miRNA-mediated gene silencing using a Drosophila S2 cell-based controllable expression system	Translational repression occurs first, followed by mRNA deadenylation and decay
4.	Giussani M [16]	2021	Retrospective Cohort: 288 plasma samples (100 malignant, 89 benign, 99 healthy). Prospective Cohort: 289 women.	Circulating miRNAs miR-625, miR-423-5p, miR-370-3p, miR-181c, and miR-301b)	To validate circulating miRNAs as novel non-invasive diagnostic biomarkers in order to distinguish malignant lesions from benign breast lesions	Liquid biopsies using circulating miRNAs are more effective than single-protein assays for early detection.
5.	Yan LX [17]	2008	113 malignant breast tumors (and controls)	miR-21 (and other dysregulated miRNAs)	To evaluate the overexpression of miR-21 in human breast cancer and the association with advanced clinical stage, lymph node metastasis and poor prognosis	Identified miR-21 as a key oncogene in breast cancer
6.	Ye X [18]	2014	HER2-positive breast cancer cell line (SK-BR-3) and xenograft mouse models	miR-221	To show that miR-221 promotes trastuzumab-resistance and metastasis in HER2-positive breast cancer by targeting PTEN	Showed that specific circulating miRNAs (like miR-21 and miR-155) can distinguish breast cancer patients from healthy controls with high sensitivity
7.	Zou R [19]	2022	663 serum samples (multiple cohorts for discovery and validation)	8-miRNA panel (circulating)	To develop and validate a circulating miRNA panel for the early detection of breast cancer across different ethnic groups	Young women with breast cancer have more aggressive molecular subtypes (Triple-Negative) and worse 5-year survival rates compared to older patients
8.	Zhao Q [20]	2022	234 subjects (120 BC, 64 other cancers, 50 controls)	miR-19b, miR-16, miR-106a (3-miRNA model)	To elaborate a highly specific miR-19b-based circulating miRNA model for the diagnosis of breast cancer	Similar outcomes as the previous study
9.	Heneghan HM [21]	2010	multiple patient cohorts analysed	Systemic miRNA-195 (and a 3-miRNA combination)	To evaluate if circulating miRNA-195 can differentiate breast cancer from other malignancies and serve as a biomarker for non-invasive and early-stage disease	miRNAs are stable in systemic circulation and can serve as non-invasive diagnostic “fingerprints” for breast cancer
10.	Liu H [22]	2022	112 breast cancer patients and 59 healthy controls	Circulating miRNA-103a-3p	To evaluate circulating miR-103a-3p as a potential diagnostic and prognostic biomarker for breast cancer	Exosomal miRNAs in liquid biopsies, concluding they provide a more protected and accurate reflection of tumor status than free-floating RNA
11.	Hannafon BN [23]	2016	N/A	Plasma exosome microRNAs	To identify a unique microRNA signature within plasma exosomes that is indicative of breast cancer	Extracellular vesicle-derived miR-21 and miR-1246 are superior plasma biomarkers for identifying breast cancer
12.	Madhavan D [24]	2016	575 samples (40 profiled, 237 validated, 335 in second cohort)	16-miRNA panel (e.g., miR-200 family and miR-210)	To identify circulating miRNAs that serve as prognostic markers in metastatic breast cancer and for the early detection of metastasis	Circulating miRNAs can predict metastatic breast cancer progression earlier than traditional imaging techniques
13.	Li H [25]	2018	HER2-positive metastatic patient cohort	Serum microRNA signature	To identify a serum microRNA signature that predicts benefit (treatment response and survival) from trastuzumab therapy in HER2-positive metastatic breast cancer	miRNA expression profiles can predict how a patient will respond to chemotherapy
14.	Anwar SL [26]	2019	102 Breast Cancer patients and 15 healthy women	Circulating MiR-21	To assess circulating miR-21 expression and its potential as a therapeutic monitoring marker and its correlation with clinical outcome	DNA methylation of miRNA genes is a primary driver of their dysregulation in tumors
15.	Todorova VK [27]	2022	20 Breast Cancer patients (treated with neoadjuvant chemotherapy)	Exosomal microRNAs (profiling by RNA sequencing)	To investigate whether circulating exosomal microRNAs could predict pathological complete response to neoadjuvant chemotherapy	A panel of multiple miRNAs is significantly more accurate for clinical diagnosis than any single miRNA biomarker alone

N/A—not applicable.

## Data Availability

No new data were created or analyzed in this study.

## Supplementary Materials

The following supporting information can be downloaded at: https://www.mdpi.com/article/10.3390/biomedicines14030502/s1, Table S1: Methodological Summary of Included Key Studies; Table S2: Appraisal of Bias (Simplified QUADAS-2).

## Abbreviations

The following abbreviations are used in this manuscript:

miRNAs	MicroRNA
TDLU	Terminal duct lobular unit
IHC	Immunohistochemistry
ER	Estrogen receptor
PR	Progesterone receptor
HER2	Human epidermal growth factor receptor 2
c-miRNAs	Circulating miRNAs
TNBC	Triple-Negative Breast Cancer
Onco-miRs	Oncogenic MicroRNAs
TS-miRs	Tumor-Suppressor MicroRNAs
PTEN	Phosphatase and Tensin Homolog
EMT	Epithelial–mesenchymal transition
CSCs	Cancer Stem-like Cells
AUC	Area Under the Curve
SFRP1	Secreted frizzled-related protein 1
RT-qPCR	Reverse Transcription quantitative PCR
NGS	Next Generation Sequencing
EVs	Extracellular Vesicles

## References

[1] Kim J., Harper A., McCormack V., Sung H., Houssami N., Morgan E., Mutebi M., Garvey G., Soerjomataram I., Fidler-Benaoudia M. (2025). Global patterns and trends in breast cancer incidence and mortality across 185 countries. Nat. Med..

[2] Makki J. (2015). Diversity of Breast Carcinoma: Histological Subtypes and Clinical Relevance. Clin. Med. Insights Pathol..

[3] Cserni G. (2020). Histological type and typing of breast carcinomas and the WHO classification changes over time. Pathologica.

[4] Watkins E.J. (2019). Overview of breast cancer. JAAPA.

[5] Tsang J.Y.S., Tse G.M. (2020). Molecular Classification of Breast Cancer. Adv. Anat. Pathol..

[6] Hammond M.E.H., Hayes D.F., Dowsett M., Allred D.C., Hagerty K.L., Badve S., Fitzgibbons P.L., Francis G., Goldstein N.S., Hayes M. (2010). American Society of Clinical Oncology/College of American Pathologists Guideline Recommendations for Immunohistochemical Testing of Estrogen and Progesterone Receptors in Breast Cancer. J. Clin. Oncol..

[7] Voduc K.D., Cheang M.C.U., Tyldesley S., Gelmon K., Nielsen T.O., Kennecke H. (2010). Breast Cancer Subtypes and the Risk of Local and Regional Relapse. J. Clin. Oncol..

[8] Parker J.S., Mullins M., Cheang M.C.U., Leung S., Voduc D., Vickery T., Davies S., Fauron C., He X., Hu Z. (2009). Supervised Risk Predictor of Breast Cancer Based on Intrinsic Subtypes. J. Clin. Oncol..

[9] Munir J., Nisha Y., Islam N., Bissell M.B., Schwarz B.A., Cordeiro E., Seely J.M. (2025). Impact of Method of Detection of Breast Cancer on Clinical Outcomes in Individuals Aged 40 Years or Older. Radiol. Imaging Cancer.

[10] Policastro B., Nissen N., Alves C.L. (2025). Deciphering Breast Tumor Heterogeneity Through Patient-Derived Organoids and Circulating Tumor Cells. J. Pers. Med..

[11] Degheidy M.S., Abou-Elalla A.A., Kamel M.M., Abdel-Ghany S., Arneth B., Sabit H. (2025). Regulatory Roles of miR-155-5p, miR-21-5p, miR-93-5p, and miR-140-5p in Breast Cancer Progression. Curr. Issues Mol. Biol..

[12] Cayrefourcq L., Alix-Panabières C. (2020). Clinical relevance of liquid biopsy in breast cancer: Update in 2020. Expert Rev. Mol. Diagn..

[13] Liu X., Papukashvili D., Wang Z., Liu Y., Chen X., Li J., Li Z., Hu L., Li Z., Rcheulishvili N. (2022). Potential utility of miRNAs for liquid biopsy in breast cancer. Front. Oncol..

[14] Agrawal P., Olgun G., Singh A., Gopalan V., Hannenhalli S. (2025). Characterizing the pan-cancer role of exosomal miRNAs in metastasis across cancers. Comput. Struct. Biotechnol. J..

[15] Djuranovic S., Nahvi A., Green R. (2012). miRNA-Mediated Gene Silencing by Translational Repression Followed by mRNA Deadenylation and Decay. Science.

[16] Giussani M., Ciniselli C.M., De Cecco L., Lecchi M., Dugo M., Gargiuli C., Mariancini A., Mancinelli E., Cosentino G., Veneroni S. (2021). Circulating miRNAs as Novel Non-Invasive Biomarkers to Aid the Early Diagnosis of Suspicious Breast Lesions for Which Biopsy Is Recommended. Cancers.

[17] Yan L.X., Huang X.F., Shao Q., Huang M.Y., Deng L., Wu Q.L., Zeng Y.X., Shao J.Y. (2008). MicroRNA miR-21 overexpression in human breast cancer is associated with advanced clinical stage, lymph node metastasis and patient poor prognosis. RNA.

[18] Ye X., Bai W., Zhu H., Zhang X., Chen Y., Wang L., Yang A., Zhao J., Jia L. (2014). MiR-221 promotes trastuzumab-resistance and metastasis in HER2-positive breast cancers by targeting PTEN. BMB Rep..

[19] Zou R., Loke S.Y., Tang Y.C., Too H.P., Zhou L., Lee A.S.G., Hartman M. (2022). Development and validation of a circulating microRNA panel for the early detection of breast cancer. Br. J. Cancer.

[20] Zhao Q., Shen L., Lü J., Xie H., Li D., Shang Y., Huang L., Meng L., An X., Zhou J. (2022). A circulating miR-19b-based model in diagnosis of human breast cancer. Front. Mol. Biosci..

[21] Heneghan H.M., Miller N., Kelly R., Newell J., Kerin M.J. (2010). Systemic miRNA-195 Differentiates Breast Cancer from Other Malignancies and Is a Potential Biomarker for Detecting Noninvasive and Early Stage Disease. Oncologist.

[22] Liu H., Bian Q.Z., Zhang W., Cui H.B. (2021). Circulating microRNA-103a-3p could be a diagnostic and prognostic biomarker for breast cancer. Oncol. Lett..

[23] Hannafon B.N., Trigoso Y.D., Calloway C.L., Zhao Y.D., Lum D.H., Welm A.L., Zhao Z.J., Blick K.E., Dooley W.C., Ding W.Q. (2016). Plasma exosome microRNAs are indicative of breast cancer. Breast Cancer Res..

[24] Madhavan D., Peng C., Wallwiener M., Zucknick M., Nees J., Schott S., Rudolph A., Riethdorf S., Trumpp A., Pantel K. (2016). Circulating miRNAs with prognostic value in metastatic breast cancer and for early detection of metastasis. Carcinogenesis.

[25] Li H., Liu J., Chen J., Wang H., Yang L., Chen F., Fan S., Wang J., Shao B., Yin D. (2018). A serum microRNA signature predicts trastuzumab benefit in HER2-positive metastatic breast cancer patients. Nat. Commun..

[26] Anwar S.L., Sari D.N.I., Kartika A.I., Fitria M.S., Tanjung D.S., Rakhmina D., Wardana T., Astuti I., Haryana S.M., Aryandono T. (2019). Upregulation of Circulating MiR-21 Expression as a Potential Biomarker for Therapeutic Monitoring and Clinical Outcome in Breast Cancer. Asian Pac. J. Cancer Prev..

[27] Todorova V.K., Byrum S.D., Gies A.J., Haynie C., Smith H., Reyna N.S., Makhoul I. (2022). Circulating Exosomal microRNAs as Predictive Biomarkers of Neoadjuvant Chemotherapy Response in Breast Cancer. Curr. Oncol..

[28] Arun R.P., Cahill H.F., Marcato P. (2022). Breast Cancer Subtype-Specific miRNAs: Networks, Impacts, and the Potential for Intervention. Biomedicines.

[29] Orrantia-Borunda E., Anchondo-Nuñez P., Acuña-Aguilar L.E., Gómez-Valles F.O., Ramírez-Valdespino C.A. (2022). Subtypes of Breast Cancer. Breast Cancer.

[30] Yan L.-J., Lau A.T.Y., Xu Y.-M. (2024). The regulation of microRNAs on chemoresistance in triple-negative breast cancer: A recent update. Epigenomics.

[31] Mathe A., Scott R., Avery-Kiejda K. (2015). miRNAs and Other Epigenetic Changes as Biomarkers in Triple Negative Breast Cancer. Int. J. Mol. Sci..

[32] Yang F., Zhang W., Shein Y., Guan X. (2015). Identification of dysregulated microRNAs in triple-negative breast cancer (Review). Int. J. Oncol..

[33] Loh H.Y., Norman B.P., Lai K.S., Rahman N.M.A., Alitheen N.B.M., Osman M.A. (2019). The Regulatory Role of MicroRNAs in Breast Cancer. Int. J. Mol. Sci..

[34] Shinde S.S., Ahmed S., Malik J.A., Hani U., Khanam A., Ashraf Bhat F., Ahmad Mir S., Ghazwani M., Wahab S., Haider N. (2023). Therapeutic Delivery of Tumor Suppressor miRNAs for Breast Cancer Treatment. Biology.

[35] Ghafouri-Fard S., Khanbabapour Sasi A., Abak A., Shoorei H., Khoshkar A., Taheri M. (2021). Contribution of miRNAs in the Pathogenesis of Breast Cancer. Front. Oncol..

[36] Wu D., Thompson L.U., Comelli E.M. (2022). MicroRNAs: A Link between Mammary Gland Development and Breast Cancer. Int. J. Mol. Sci..

[37] Humphries B., Wang Z., Yang C. (2021). MicroRNA Regulation of Breast Cancer Stemness. Int. J. Mol. Sci..

[38] Zhang J., Ma L. (2012). MicroRNA control of epithelial–mesenchymal transition and metastasis. Cancer Metastasis Rev..

[39] Fogazzi V., Kapahnke M., Cataldo A., Plantamura I., Tagliabue E., Di Cosimo S., Cosentino G., Iorio M.V. (2022). The Role of MicroRNAs in HER2-Positive Breast Cancer: Where We Are and Future Prospective. Cancers.

[40] Muluhngwi P., Klinge C.M. (2015). Roles for miRNAs in endocrine resistance in breast cancer. Endocr. Relat. Cancer.

[41] Singh S., Saini H., Sharma A., Gupta S., Huddar V.G., Tripathi R. (2023). Breast cancer: miRNAs monitoring chemoresistance and systemic therapy. Front. Oncol..

[42] Confuorti C., Jaramillo M., Plante I. (2024). Hormonal regulation of miRNA during mammary gland development. Biol. Open..

[43] Muñoz J.P., Pérez-Moreno P., Pérez Y., Calaf G.M. (2023). The Role of MicroRNAs in Breast Cancer and the Challenges of Their Clinical Application. Diagnostics.

[44] Svoronos A.A., Engelman D.M., Slack F.J. (2016). OncomiR or Tumor Suppressor? The Duplicity of MicroRNAs in Cancer. Cancer Res..

[45] Baylie T., Kasaw M., Getinet M., Getie G., Jemal M., Nigatu A., Ahmed H., Bogale M. (2024). The role of miRNAs as biomarkers in breast cancer. Front. Oncol..

[46] Jelski W., Okrasinska S., Mroczko B. (2025). microRNAs as Biomarkers of Breast Cancer. Int. J. Mol. Sci..

[47] Chakrabortty A., Patton D.J., Smith B.F., Agarwal P. (2023). miRNAs: Potential as Biomarkers and Therapeutic Targets for Cancer. Genes.

[48] Sundarbose K., Kartha R., Subramanian S. (2013). MicroRNAs as Biomarkers in Cancer. Diagnostics.

[49] Pająk W., Kleinrok J., Pec J., Michno K., Wojtas J., Badach M., Teresińska B., Baj J. (2025). Micro RNA in Colorectal Cancer—Potential Diagnostic and Prognostic Markers—An Updated Review. Int. J. Mol. Sci..

[50] Vo T.H., EL-Sherbieny Abdelaal E., Jordan E., O’Donovan O., McNeela E.A., Mehta J.P., Rani S. (2024). miRNAs as biomarkers of therapeutic response to HER2-targeted treatment in breast cancer: A systematic review. Biochem. Biophys. Rep..

[51] Cardinali B., Tasso R., Piccioli P., Ciferri M.C., Quarto R., Del Mastro L. (2022). Circulating miRNAs in Breast Cancer Diagnosis and Prognosis. Cancers.

[52] Bautista-Sánchez D., Arriaga-Canon C., Pedroza-Torres A., De La Rosa-Velázquez I.A., González-Barrios R., Contreras-Espinosa L., Montiel-Manríquez R., Castro-Hernández C., Fragoso-Ontiveros V., Álvarez-Gómez R.M. (2020). The Promising Role of miR-21 as a Cancer Biomarker and Its Importance in RNA-Based Therapeutics. Mol. Ther. Nucleic Acids.

[53] Prasad M., Hamsa D., Fareed M., Karobari M.I. (2025). An update on the molecular mechanisms underlying the progression of miR-21 in oral cancer. World J. Surg. Oncol..

[54] Wei X., Xiong X., Chen Z., Chen B., Zhang C., Zhang W. (2025). MicroRNA155 in non-small cell lung cancer: A potential therapeutic target. Front. Oncol..

[55] Shah N.R. (2014). MicroRNAs in pathogenesis of breast cancer: Implications in diagnosis and treatment. World J. Clin. Oncol..

[56] Otmani K., Lewalle P. (2021). Tumor Suppressor miRNA in Cancer Cells and the Tumor Microenvironment: Mechanism of Deregulation and Clinical Implications. Front. Oncol..

[57] Subramanian K., Sinha R. (2024). Functions of Differentially Regulated miRNAs in Breast Cancer Progression: Potential Markers for Early Detection and Candidates for Therapy. Biomedicines.

[58] Jang J., Kim Y., Kang K., Kim K., Park Y., Kim C. (2020). Multiple microRNAs as biomarkers for early breast cancer diagnosis. Mol. Clin. Oncol..

[59] Song Q., Zhang Y., Liu H., Du Y. (2020). Potential of Using Cell-Free DNA and miRNA in Breast Milk to Screen Early Breast Cancer. Biomed. Res. Int..

[60] Erbes T., Hirschfeld M., Rücker G., Jaeger M., Boas J., Iborra S., Mayer S., Gitsch G., Stickeler E. (2015). Feasibility of urinary microRNA detection in breast cancer patients and its potential as an innovative non-invasive biomarker. BMC Cancer.

[61] Ban E., Chae D.K., Yoo Y.S., Song E.J. (2017). An improvement of miRNA extraction efficiency in human plasma. Anal. Bioanal. Chem..

[62] Alimirzaie S., Bagherzadeh M., Akbari M.R. (2019). Liquid biopsy in breast cancer: A comprehensive review. Clin. Genet..

[63] Quirico L., Orso F. (2020). The power of microRNAs as diagnostic and prognostic biomarkers in liquid biopsies. Cancer Drug Resist..

[64] McDonald J.S., Milosevic D., Reddi H.V., Grebe S.K., Algeciras-Schimnich A. (2011). Analysis of Circulating MicroRNA: Preanalytical and Analytical Challenges. Clin. Chem..

[65] Wu H.J., Chu P.Y. (2022). Current and Developing Liquid Biopsy Techniques for Breast Cancer. Cancers.

[66] Duque G., Manterola C., Otzen T., Arias C., Galindo B., Mora M., Guerrero E., García N. (2022). Clinical utility of liquid biopsy in breast cancer: A systematic review. Clin. Genet..

[67] Fischer C., Deutsch T.M., Feisst M., Rippinger N., Riedel F., Hartkopf A.D., Brucker S.Y., Domschke C., Fremd C., Michel L. (2022). Circulating miR-200 family as predictive markers during systemic therapy of metastatic breast cancer. Arch. Gynecol. Obstet..

[68] Alba-Bernal A., Lavado-Valenzuela R., Domínguez-Recio M.E., Jiménez-Rodriguez B., Queipo-Ortuño M.I., Alba E., Comino-Méndez I. (2020). Challenges and achievements of liquid biopsy technologies employed in early breast cancer. eBioMedicine.

[69] Oshi M., Murthy V., Takahashi H., Huyser M., Okano M., Tokumaru Y., Rashid O.M., Matsuyama R., Endo I., Takabe K. (2021). Urine as a Source of Liquid Biopsy for Cancer. Cancers.

[70] Wang J., Sijing S., Jie Z., Guinian W. (2017). Prognostic value of circulating microRNA-21 for breast cancer: A systematic review and meta-analysis. Artif. Cells Nanomed. Biotechnol..

