# Supplementary material for: MicroRNAs in Breast Cancer: Diagnostic and Prognostic Potential, Challenges, and Clinical Reliability
Source: Biomedicines. 2026 Feb 25;14(3):502. doi: 10.3390/biomedicines14030502 (PMC13024443; doi:10.3390/biomedicines14030502)
Supplement: Supplementary file 1 [file biomedicines-14-00502-s001.zip › biomedicines-4119783-supplementary.pdf]

Supplementary Materials

Element, Description

Databases, PubMed and Google Scholar from 2008 onwards

Search Date,"[January 1, 2008 to December 31, 2025]."

Inclusion Criteria:

1. Human subjects with confirmed Breast Cancer.

2. Original research reporting diagnostic/prognostic metrics (AUC, Sen, Spe).

3. Minimum cohort size: n≥50.

4. Use of validated platforms (RT-qPCR, NGS, ddPCR).

Exclusion Criteria:

1. Cell-line or animal-only studies.

2. Reviews, editorials, or conference abstracts.

3. Studies lacking an independent validation cohort.

4. Non-English language."

Screening Process: Two independent reviewers screened titles/abstracts. Discrepancies were resolved by a third senior reviewer.

Table S1. Methodological Summary of Included Key Studies

Lead Author	Year	Sample Source	Platform	Cohort (Disc / Val)	Metric (AUC/Sen/Spe)	Validation Type
Heneghan HM	2010	Whole Blood	RT-qPCR	n=148/20	AUC:0.91	Internal Cross-Val
Madhavan D	2012	Plasma	RT-qPCR	n=120/82	AUC:0.70–0.88	Independent Cohort
Hannafon BN	2016	Exosomes	NGS	n=32/24	Sen:91%/Spe:82%	External Validation
Anwar SL	2013	Plasma	RT-qPCR	n=112/112	AUC:0.92	Technical Validation
Zou R	2011	Serum	RT-qPCR	n=120/60	AUC:0.95	Blinded

						Validation
Todorova VK	2019	Plasma	NGS/qPCR	n=46/20	AUC:0.85	Independent Cohort

Table S2. Appraisal of Bias (Simplified QUADAS-2)

Study	Patient Selection	Index Test	Reference Standard	Flow & Timing	Overall Quality
Agrawal P	Low Risk	Low Risk	Low Risk	Low Risk	High
Degheidy MS	High Risk*	Low Risk	Low Risk	Low Risk	Moderate
Ye X	Low Risk	High Risk**	Low Risk	Low Risk	Moderate

*High Risk in Patient Selection usually means using "healthy" volunteers instead of patients with benign breast disease as controls.

**High Risk in Index Test usually means the threshold for the miRNA panel was not pre-specified.
